# Functional Magnetic Resonance Imaging Correlates of Cognitive Reappraisal of Negative Emotion: A Brain Activation and Generalized Psychophysiological Interaction Study

**DOI:** 10.1002/brb3.71821

**Published:** 2026-10-08

**Authors:** Yuluo Liu, Shan Gui, Longda Ma, Lian Yang, Zilong Liu, Yiwu Zhou, Xiangyu Wang

**Affiliations:** ^1^ Department of Forensic Science Guangdong Police College Guangzhou People's Republic of China; ^2^ Department of Radiology, Daping Hospital Army Medical University Daping Chongqing People's Republic of China; ^3^ Department of Forensic Medicine, Tongji Medical College Huazhong University of Science and Technology Wuhan People's Republic of China; ^4^ Department of Radiology, Wuhan Union Hospital, Tongji Medical College Huazhong University of Science and Technology Wuhan People's Republic of China; ^5^ Department of Basic Medical Science Guangzhou Health Science College Guangzhou People's Republic of China

**Keywords:** brain activation, cognitive reappraisal, emotion regulation, fMRI, psychophysiological interaction

## Abstract

**Background:**

Cognitive reappraisal has been extensively investigated using fMRI, yet the neural mechanisms underlying up‐ versus down‐regulation of negative emotion remain incompletely understood. This study aimed to examine brain activation and task‐dependent functional connectivity for both regulatory goals.

**Methods:**

A well‐established cognitive reappraisal task was employed during an fMRI scan, with 21 participants instructed to up‐ and down‐regulate negative emotions by mentally reinterpreting the stimuli. Whole‐brain activation analyses included a conjunction‐null analysis and differential contrasts to identify shared and goal‐specific activation. Generalized psychophysiological interaction (gPPI) analyses were also performed to examine task‐dependent functional connectivity.

**Results:**

Down‐regulation primarily engaged the ventrolateral prefrontal cortex (VLPFC) and middle temporal gyrus (MTG), whereas up‐regulation recruited prefrontal regions and the left insula. The conjunction‐null analysis revealed common activation in a predominantly left‐lateralized frontoparietal–medial frontal network beyond viewing negative pictures. Differential contrasts showed that up‐regulation specifically recruited the left postcentral gyrus, whereas down‐regulation specifically recruited occipital regions and the right dorsolateral prefrontal cortex (DLPFC). In exploratory and uncorrected gPPI analyses, during down‐regulation, the VLPFC was positively coupled with the right MTG, whereas during up‐regulation, the left DLPFC was coupled with the left insula and dorsal striatum.

**Conclusion:**

These findings suggest that up‐ and down‐regulation share a common frontoparietal–medial frontal network, but differ in goal‐specific activation and connectivity patterns. Down‐regulation may rely more on cognitive and semantic processing, whereas up‐regulation may involve reward‐related and interoceptive mechanisms. Given the exploratory nature of the gPPI findings, the connectivity results should be considered preliminary.

## Graphical abstract

Neural activation and task‐dependent functional connectivity associated with cognitive reappraisal of negative emotion. Up‐ and down‐regulation shared a predominantly left‐lateralized frontoparietal‐medial frontal network but showed goal‐specific patterns of activation and connectivity. Exploratory, uncorrected generalized psychophysiological interaction (gPPI) findings suggest distinct connectivity patterns associated with the two regulatory goals. DLPFC, dorsolateral prefrontal cortex; VLPFC, ventrolateral prefrontal cortex; MTG, middle temporal gyrus.

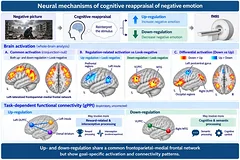

## 1 Introduction

People frequently encounter events that trigger negative emotions in daily social life (Gross et al. 2006; Berking and Wupperman 2012; Berkman and Lieberman 2009; Gross and Muñoz 1995). Negative emotions can severely affect our daily life and work, as well as harm our physical and mental health (Gross 1998). Therefore, regulating emotions is crucial for adapting to various situations. Emotion regulation refers to the ability to experience, influence, and express emotions (Gross 1998). The primary emotion regulation strategies include attentional deployment, cognitive reappraisal, and expressive suppression. Among these strategies, cognitive reappraisal is considered one of the most effective means of reducing negative emotions (Gross 2002). Cognitive reappraisal involves cognitively changing the meaning of a situation to alter its emotional impact. In addition, cognitive reappraisal can be further categorized into self‐focused and situation‐focused methods. Self‐focused reappraisal, also known as “detachment” or “distancing”, involves participants being instructed to decrease or increase their emotional response by altering its personal relevance. In contrast, situation‐focused reappraisal, also called “reinterpretation”, involves participants being instructed to change their perspective on a situation by imagining its positive or negative outcomes. Psychological models suggest that cognitive reappraisal involves various cognitive control processes, including attention, memory, and thought regulation (Ochsner and Gross 2008). Despite extensive research, however, the precise neural mechanisms underlying cognitive reappraisal, particularly in the context of comparing up‐ versus down‐regulation of negative emotion, are not yet fully understood.

Numerous neuroimaging studies have identified a core brain network engaged during cognitive reappraisal. Influential meta‐analyses have consistently pointed to the dorsolateral prefrontal cortex (DLPFC), ventrolateral prefrontal cortex (VLPFC), dorsomedial prefrontal cortex (DMPFC), dorsal anterior cingulate cortex (dACC), temporal gyrus, and inferior parietal lobule as key nodes (Morawetz et al. 2017; Buhle et al. 2014; Kohn et al. 2014; McRae et al. 2010). In line with the cognitive control model of emotion, some studies have shown that DMPFC and VLPFC reduce amygdala responses via their influence on the ventromedial prefrontal cortex (VMPFC) during cognitive reappraisal (Urry et al. 2006; Johnstone et al. 2007; Ochsner et al. 2012). However, most of these studies have focused almost exclusively on down‐regulation of negative emotion. By contrast, up‐regulation has received much less attention, despite its adaptive value in empathy, threat anticipation, and risk assessment. Clinically, the inability to upregulate negative emotion is a core feature of schizophrenia and depression, while excessive upregulation contributes to depressive rumination (Yoon et al. 2013). Among the few studies that have directly compared up‐ and down‐regulation, the findings remain limited and somewhat heterogeneous (Urry et al. 2006; Morawetz et al. 2016; Urry et al. 2009; Li et al. 2018; Kim and Hamann 2007). To clarify these inconsistent findings, a large‐scale coordinate‐based meta‐analysis was conducted, revealing that both up‐ and down‐regulation reliably activate the bilateral VLPFC, DMPFC, and supplementary motor area (SMA), suggesting a shared prefrontal core network for emotion regulation (Morawetz et al. 2017). However, goal‐specific activations were also identified: down‐regulation more strongly engaged the right DLPFC and right inferior parietal lobule, whereas up‐regulation preferentially activated the left insula and SMA (Morawetz et al. 2017). These findings further indicate that the two goals recruit partially distinct brain regions at the activation level. These meta‐analytic findings provide a useful synthesis of activation‐level evidence, though the contrasts were derived from between‐study comparisons rather than within‐subject direct contrasts. Moreover, activation‐level meta‐analyses cannot reveal how these regions interact with each other or with subcortical emotion‐processing regions.

To capture these interactions, task‐dependent functional connectivity analyses, specifically the generalized psychophysiological interaction (gPPI) approach, are required (McLaren et al. 2012). The gPPI indexes task‐induced changes in inter‐regional coupling and permits simultaneous modeling of multiple task conditions within a single framework, making it particularly valuable for investigating dynamic affective processes such as emotion regulation. A recent coordinate‐based meta‐analysis of 15 PPI studies illustrates this trend, reporting convergent amygdala–prefrontal connectivity, including the right DLPFC, left VLPFC, and DMPFC, during down‐regulation via reappraisal (Berboth and Morawetz 2021). However, these findings were derived from studies that have overwhelmingly targeted down‐regulation, whereas up‐regulation and direct comparisons remain far less explored. Consequently, it remains unclear whether up‐ and down‐regulation engage shared or goal‐specific coupling patterns within the emotion‐regulation network.

These unresolved questions motivated the present study, in which we employed a within‐subject fMRI design integrating whole‐brain mapping with gPPI connectivity analysis to directly contrast up‐ versus down‐regulation of negative emotion. Specifically, we aimed to identify common and distinct activation patterns for the two goals, and to characterize the differential task‐dependent functional coupling between prefrontal control regions and emotion‐processing regions. By directly comparing the two regulatory goals within the same cohort and combining activation with connectivity measures, this study extends prior work beyond both regional activation and the exclusive focus on down‐regulation.

## 2 Materials and Methods

### 2.1 Participants

Twenty‐two volunteers were initially recruited for this experiment via paper flyers and electronic posters from the campus community in Tongji Medical College, Huazhong University of Science and Technology. All participants were right‐handed, had normal or corrected‐to‐normal vision, and reported no history of any psychiatric, medical, or neurological illness. The study was approved by the Human Ethics Committee for Human Research in Tongji Medical College (approval number: 2026S096). All methods were carried out in accordance with the Declaration of Helsinki and relevant guidelines and regulations. Informed consent was obtained from all participants for study participation. Informed consent for publication of identifying information in an open‐access publication was also obtained from all participants. One participant was excluded due to excessive head motion (maximal displacement > 3 mm), leaving a final sample of 21 participants (mean age = 26.1 years, SD = 3.2). Participants also completed a battery of self‐report questionnaires assessing personality traits and emotion regulation for sample characterization (Yang et al. 2018; Qian et al. 2018; Zhang 2008; Gratz and Roemer 2004). No prespecified cutoff scores were applied, and no participants were excluded based on these measures. The demographic characteristics of the participants are presented in Table 1. The sample size (*n = *21) was primarily determined by practical constraints, including the high cost of fMRI scanning and the stringent inclusion criteria. No a priori power analysis was conducted. The sample size is comparable to that of several previous task‐fMRI studies of cognitive reappraisal (Urry et al. 2006; Morawetz et al. 2016; Urry et al. 2009; Kim and Hamann 2007; Holland and Kensinger 2013). The within‐subject design, in which each participant served as their own control across up‐regulation, down‐regulation, and look conditions, enhances statistical power by reducing inter‐individual variability.

**TABLE 1:** Demographic characteristics of the participants.

Variable	Participants (mean (SD))
N (female)	21 (9)
Age (years)	26.1 (3.2)
Level of education^a^	5.7 (0.5)
Self‐reported handness (percentage right‐handed)	100%

Abbreviation: SD = standard deviation.

^a^Assessed by the International Standard Classification of Education (ISCED).

### 2.2 Stimuli and Task Design

Stimuli for the reappraisal task were negative and neutral pictures chosen from the International Affective Picture System (IAPS) (Lang et al. 1993). The valence, arousal, and dominance ratings of these pictures had been systematically assessed in Chinese participants (Liu and Xu 2009). Thirty negative pictures [three sets with each set containing 10 pictures (Set 1: valence, 2.52 ± 0.34, arousal, 6.08 ± 0.98; Set 2: valence, 2.38 ± 0.52, arousal, 5.81 ± 0.60; Set 3: valence, 2.31 ± 0.53, arousal, 6.01 ± 0.89)] and 10 neutral pictures (valence, 5.36 ± 0.29, arousal, 3.65 ± 0.89) were selected with no repetition, giving a total of 40 stimuli. Independent‐samples t tests confirmed significant differences between negative and neutral pictures in both valence (mean difference = ‐2.96, t(38) = ‐18.92, *p < *0.001, Cohen's d = ‐6.91, 95% CI [−3.28, −2.64]) and arousal (mean difference = 2.32, t(38) = 7.62, *p < *0.001, Cohen's *d* = 2.78, 95% CI [1.71, 2.94]). Furthermore, one‐way ANOVAs revealed that the three sets of negative pictures did not differ significantly from one another in either valence (F(2, 27) = 0.52, *p = *0.598) or arousal (F(2, 27) = 0.27, *p = *0.769). The three sets of negative pictures were pseudorandomized and counterbalanced across the three conditions (“Look”, “Down”, and “Up”) to ensure each image appeared equally often in each condition. Twenty‐four additional pictures were selected for the practice task (Li et al. 2018).

The task consisted of 40 trials, divided into 10 blocks of 4 trials each. The trial structure and timings are presented in Figure 1. Each trial comprised 1–3 s of cross fixation, 2 s of cues (“Look,” “Down,” and “Up”), 7 s of picture presentation, and 4 s of emotion rating. All cues and instructions were presented in Mandarin Chinese, the participants’ native language. In the experiment, the participants were asked to cognitively reappraise the meaning of presented pictures by situation‐focused methods according to the cues (Li et al. 2018). For the up‐negative condition, participants were instructed to imagine a more negative outcome associated with the depicted scene (e.g., worsening the situation or focusing on threatening aspects). For the down‐negative condition, they were instructed to imagine a less negative or positive outcome (e.g., reinterpreting the situation in a less threatening way). For the look‐negative and look‐neutral conditions, they were instructed to simply view the picture without engaging in any reappraisal strategy. Participants then rated their negative emotion intensity by pressing a button corresponding to a 5‐point scale (1 = “not at all negative” to 5 = “extremely negative”). Those who failed to respond on more than five trials were excluded from the analyses. The trial order was pseudorandomized and fixed across participants, with the constraints that across the entire run, no more than three negative pictures and no more than two regulation trials (up or down) appeared in succession. Although the fixed pseudorandom sequence may introduce order effects, the within‐subject design and counterbalancing partially mitigate this concern. After the scan, participants rated the valence and arousal of each picture using the Self‐Assessment Manikin (SAM) as a supplementary task (Lang 1980). Prior to scanning, participants completed a training session using 24 practice images not used in the main task. They verbalized their reappraisals aloud and received corrective feedback until they demonstrated correct application of both strategies.

**FIGURE 1:**
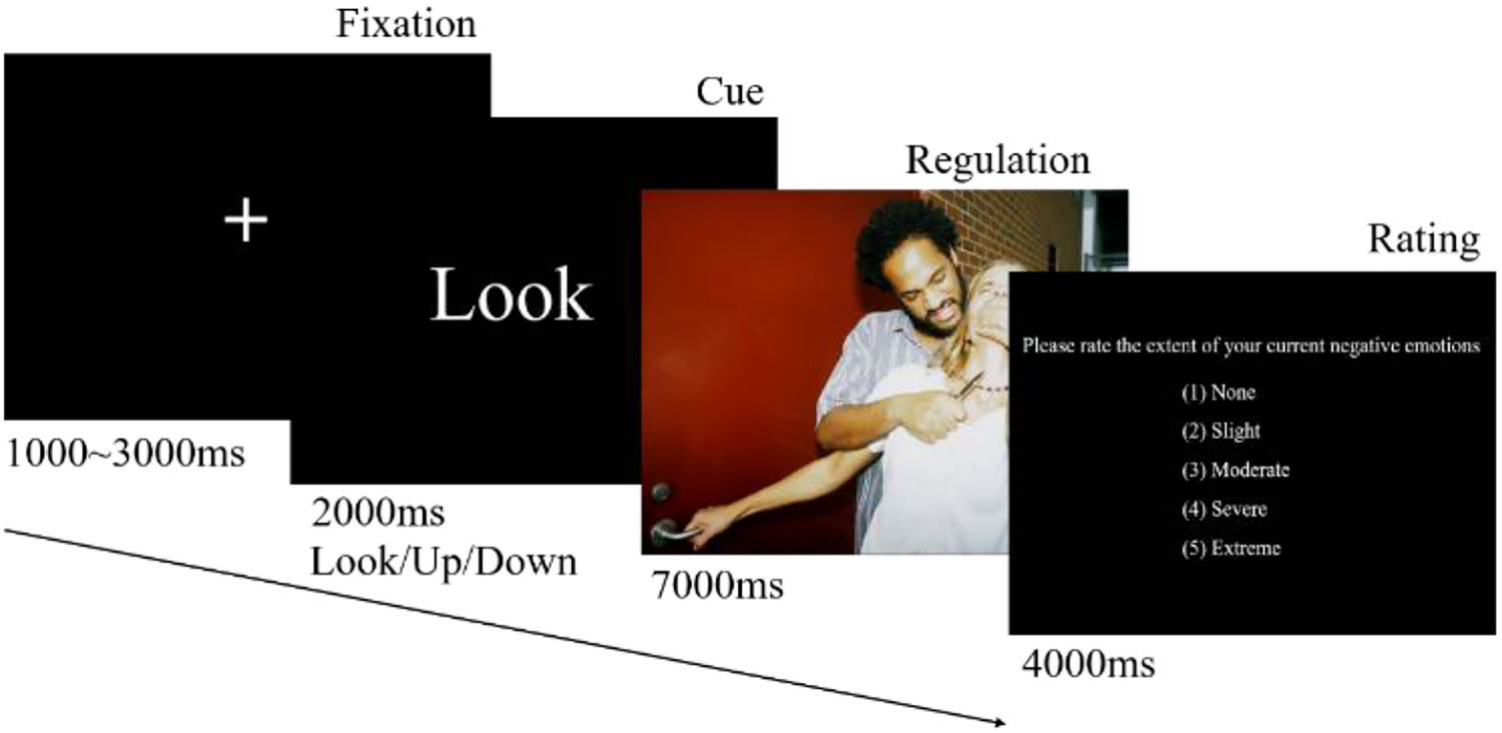
Schematic illustration of the trial procedures used in the present study. Each trial consisted of the following sequence: a 1–3 s cross fixation, a 2‐s cue (Look, Up, or Down), a 7‐s presentation of a picture (either negative or neutral), and a 4‐s rating period. During the regulation phase, participants were instructed to engage in cognitive reappraisal of the meaning of the presented pictures according to the cue. The “Look” cue instructed participants to view the picture and respond naturally. The “Up” cue instructed participants to imagine a more negative outcome, whereas the “Down” cue instructed them to imagine a less negative or positive outcome. During the rating period, participants used a 5‐button hand pad to indicate their emotional response to the picture after following the cue.

### 2.3 Image Acquisition

As participants performed the reappraisal task (Figure 1), blood oxygenation level‐dependent (BOLD) images were obtained using a Philips Ingenia CX 3T MRI scanner (Philips Medical Systems, Best, the Netherlands) with a gradient‐echo echoplanar imaging (GR‐EPI) sequence parallel to the bicommissural plane. The scanning parameters were as follows: repetition time (TR) = 800 ms, echo time (TE) = 30 ms, flip angle = 52°, voxel size = 2.4 × 2.4 × 2.4 mm^3^, field of view (FOV) = 200 × 200 mm, acquisition matrix = 84 × 83, 60 interleaved axial slices, and 752 volumes. For anatomical localization, a T1‐weighted 3D fast field echo (T1W‐3D‐TFE) structural image was acquired in the sagittal plane parallel to the bicommissural plane with the following parameters: voxel size = 0.7 × 0.7 × 0.7 mm^3^, 257 slices, TR = 11 ms, TE = 5.3 ms, flip angle = 8°, slice thickness = 0.7 mm, matrix = 368 × 368, and FOV = 256 × 256 mm (Li et al. 2018).

### 2.4 FMRI Analysis

#### 2.4.1 Preprocessing

Preprocessing was carried out using DPABI (a toolbox for Data Processing and Analysis for Brain Imaging) software (Yan et al. 2016), while the statistical analyses were conducted on SPM12 (Wellcome Trust Centre for Neuroimaging, London, UK). Slice timing correction was performed to account for the interleaved slice acquisition order (ascending, odd slices first), using the middle slice as the reference. Head motion was corrected using a six‐parameter rigid‐body transformation. Mean framewise displacement (FD) was calculated according to Power et al. (2012). Across all participants, the mean FD was 0.148 mm (SD = 0.036), indicating good overall motion control. One participant was excluded due to excessive head motion (maximal displacement > 3 mm). No volume‐level censoring (scrubbing) was performed, as all remaining participants showed acceptable head motion throughout the scanning session. Susceptibility distortion correction was not performed, as no fieldmap or reverse phase‐encoded images were acquired. The structural image was coregistered to the mean functional image after motion correction, and then segmented into gray matter, white matter, and cerebrospinal fluid using a unified segmentation algorithm. The motion‐corrected functional volumes were normalized to the MNI space (2 × 2 × 2 mm^3^ voxel size) using the normalization parameters estimated during segmentation. Finally, the normalized images were smoothed with a 6 mm full‐width at half‐maximum (FWHM) Gaussian kernel (Li et al. 2018).

#### 2.4.2 First‐Level Analysis

Individual‐level statistical analyses were performed using the general linear model (GLM) as implemented in SPM12. For each participant, four task conditions were modeled as regressors of interest: look‐neutral, look‐negative, up‐negative, and down‐negative. Each condition was modeled as a 7‐second boxcar function based on the regulation period, convolved with the canonical hemodynamic response function (HRF), with onsets defined at picture onset. The cue period (2 s) was not modeled as a separate regressor. The rating period (4 s) was modeled as a separate regressor of no interest to account for motor responses. Six head motion parameters (three translations and three rotations) were included as nuisance covariates. A 128‐s high‐pass filter was applied to remove low‐frequency drift, and temporal autocorrelation was modeled using an AR(1) process. No temporal derivatives were included in the model. No parametric modulators were included. For each participant, contrast images were generated for the following pairwise comparisons: down‐negative versus look‐negative, look‐negative versus down‐negative, up‐negative versus look‐negative, and look‐negative versus up‐negative. To examine differential neural activity between the two regulation goals, we defined direct comparisons between up‐ and down‐regulation (down‐negative versus up‐negative and up‐negative versus down‐negative). All contrast images were entered into group‐level random‐effects analyses (Li et al. 2018).

#### 2.4.3 Activation Analysis

At the second level, a one‐sample *t*‐test was first performed to examine the look‐negative versus look‐neutral contrast, confirming the emotional effect of the picture material. Additional one‐sample *t*‐tests were then conducted for the contrasts of down‐ versus look‐negative, look‐ versus down‐negative, up‐ versus look‐negative, and look‐ versus up‐negative, to identify brain regions showing activation and deactivation during up‐ and down‐regulation. In addition, to identify brain regions commonly activated during up‐ and down‐regulation beyond viewing negative pictures, we performed a conjunction‐null analysis using SPM12's minimum statistic approach (Nichols et al. 2005). The constituent contrasts were the first‐level contrast images for up‐ versus look‐negative and down‐ versus look‐negative. Each constituent contrast was thresholded at voxel‐level *p < *0.001 uncorrected as a cluster‐forming threshold, and results were considered significant at cluster‐level family‐wise error (FWE) corrected *p < *0.05. Conjunction regions were defined as voxels that survived this threshold in both constituent contrasts. The contrast down‐negative versus up‐negative was used to identify differential neural activation between up‐ and down‐regulation. Age and sex were included as covariates of no interest in all second‐level models. For the one‐sample t tests and differential contrasts, results were considered significant at cluster‐level FWE‐corrected *p < *0.05, with an initial cluster‐forming threshold of voxel‐level *p < *0.001 uncorrected (Li et al. 2018).

#### 2.4.4 Generalized Psychophysiological Interaction Analysis

To investigate task‐dependent functional connectivity, the generalized psychophysiological interaction (gPPI version 13.1) software was used for further analysis (McLaren et al. 2012; Friston et al. 1997). Seed regions were defined as 6 mm‐radius spheres centered at coordinates derived from the group‐level activation peaks in the down‐negative versus look‐negative and up‐negative versus look‐negative contrasts (Table 2). For each seed, the first eigenvariate of the BOLD time series was extracted for each participant, and neural activation in the seed region was estimated via hemodynamic deconvolution (physiological term). The estimated neural activation was multiplied with task vectors and reconvolved with a canonical HRF (PPI term). The individual task vectors were also convolved with a canonical HRF (psychological term). In the gPPI first‐level model, separate task regressors and interaction terms were formed for each of the four task conditions (look‐neutral, look‐negative, up‐negative, and down‐negative). The rating period was modeled as an additional regressor of no interest and was not included in the contrast analyses. For the gPPI contrasts, only the look‐negative, up‐negative, and down‐negative conditions were included, with look‐negative serving as the baseline condition to examine task‐dependent functional connectivity associated with emotion regulation. The look‐neutral condition was not included in the contrast analyses, as the focus was on regulation‐related connectivity relative to look‐negative. For each seed region, a first‐level GLM analysis was performed with the PPI terms, the task regressors (psychological term), the seed region time course (physiological term), and a constant term (McLaren et al. 2012). For each seed, contrasts were defined for the corresponding regulation condition versus look‐negative, as well as their reverse contrasts (e.g., down‐negative > look‐negative and up‐negative > look‐negative; look‐negative > down‐negative and look‐negative > up‐negative). One‐sample t tests were then performed at the group level for each contrast. Results were thresholded at *p < *0.001 uncorrected with a minimum cluster extent of k > 20 voxels. This threshold was chosen based on previous gPPI studies that have adopted similar exploratory cluster extents to balance sensitivity and specificity in the absence of a priori connectivity hypotheses (Li et al. 2018; McLaren et al. 2012; Lee et al. 2012; Banks et al. 2007). No correction for multiple comparisons was applied across seeds or contrasts, as the primary aim of the connectivity analyses was exploratory. When cluster‐level FWE correction was applied at this voxel threshold, no clusters survived, likely due to the modest sample size and the generally smaller effect sizes of connectivity measures compared to activation measures. Therefore, all gPPI findings are presented as exploratory and hypothesis‐generating, and should be interpreted with appropriate caution.

**TABLE 2:** Seed regions for gPPI analysis.

Seed region	Peak MNI coordinate		
	X	Y	Z
Down > look			
L Inf. Frontal G. /VLPFC	−52	22	−12
R Inf. Frontal G./VLPFC	42	26	−14
R Mid. Frontal G./DLPFC	50	16	40
L Sup. Frontal G./RLPFC	−18	54	16
Up>look			
L Inf. Frontal G./DLPFC	−54	22	20
L Sup. Frontal G./DMPFC	−4	30	52

Abbreviations: G. = gyrus; Inf. = inferior; L = left; Mid. = middle; MNI = Montreal Neurological Institute; R = right; Sup. = superior.

## 3 Results

### 3.1 Behavioral Results

#### 3.1.1 Subject Ratings of Negative Emotion

The paired t‐test revealed that negative pictures evoked significantly higher levels of negative emotion compared to neutral pictures (mean difference = −1.52, t(20) = −8.55, *p < *0.001, Cohen's *d* = −1.87, 95% CI [−1.90, −1.15]). A one‐way repeated‐measures ANOVA was conducted on trial‐wise averages of the negative emotion ratings, which revealed that cognitive reappraisal significantly altered emotional states (Greenhouse–Geisser corrected: F(1.49, 29.87) = 31.74, *p < *0.001, η^2^ *p = *0.61). Post‐hoc pairwise comparisons with Bonferroni correction showed that negative emotion ratings were significantly higher in the up‐negative condition than in the look‐negative condition (mean difference = 0.68, *p < *0.001, 95% CI [0.41, 0.95]), and significantly lower in the down‐negative condition than in the look‐negative condition (mean difference = −0.56, *p = *0.009, 95% CI [−1.00, −0.13]) (Figure 2A). These results confirm that cognitive reappraisal was effective in this experiment, with participants successfully decreasing or increasing their negative emotions as instructed.

**FIGURE 2:**
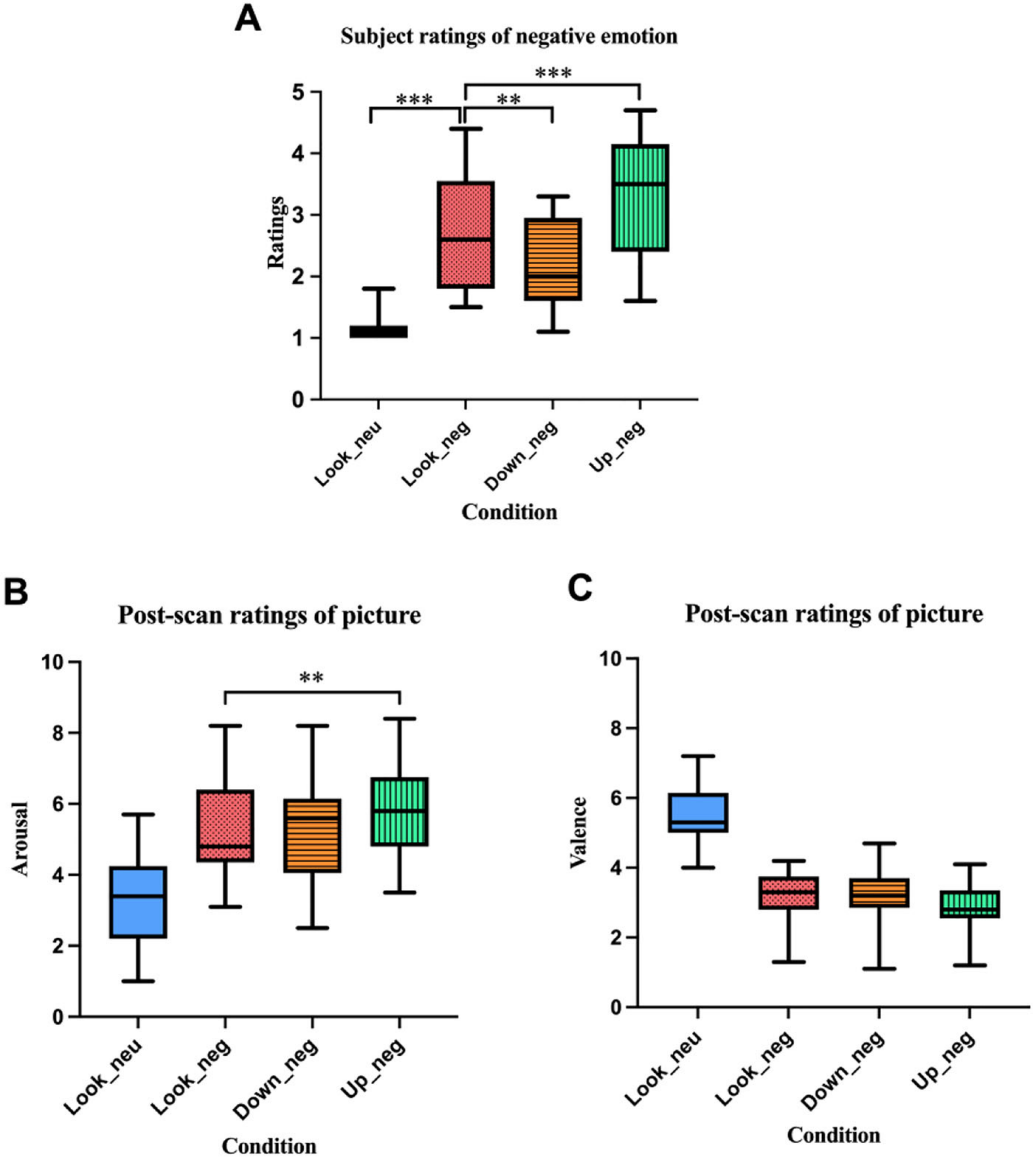
Behavioral results. (A) Subject ratings of negative emotion. Negative pictures elicited significantly higher levels of negative emotion, and participants’ emotional responses were significantly altered (either up‐ or down‐regulation) according to the given instructions. (B and C) Post‐scan ratings of the pictures. For arousal (B), the pictures used in the up‐negative condition were rated significantly higher than the look‐negative condition, whereas the pictures used in the down‐negative condition did not differ significantly from the look‐negative condition. For valence (C), the main effect was significant, but neither up‐negative nor down‐negative differed significantly from look‐negative. **p < 0.01, ***p < 0.001.

#### 3.1.2 Post‐Scan Ratings of Pictures

For the post‐scan ratings, the mean arousal ratings for the look‐negative (M = 5.24, SD = 1.44), down‐negative (M = 5.17, SD = 1.49), and up‐negative (M = 5.80, SD = 1.26) conditions showed a significant main effect of condition (F(2,40) = 10.93, *p < *0.001, η^2^ *p = *0.35). Bonferroni‐corrected pairwise comparisons showed significantly higher ratings in the up‐negative than in the look‐negative condition (mean difference = 0.55, *p = *0.008, 95% CI [0.13, 0.97]), whereas the down‐negative condition did not differ significantly from the look‐negative condition (mean difference = −0.07, *p = *1.000, 95% CI [−0.39, 0.25]). For valence (look‐negative: M = 3.16, SD = 0.74; down‐negative: M = 3.20, SD = 0.80; up‐negative: M = 2.87, SD = 0.71), a significant main effect was also found (F(2,40) = 5.52, *p = *0.008, η^2^ *p = *0.22), but no pairwise comparison survived Bonferroni correction (all *p* > 0.05). Together, these post‐scan ratings suggest that the regulation conditions may have influenced subsequent evaluations of the pictures, especially arousal, although these ratings may also reflect carry‐over effects rather than intrinsic differences between negative picture sets (Figure 2B,C).

### 3.2 fMRI Results

#### 3.2.1 Brain Activation Analysis

The results indicated that the bilateral VLPFC, superior temporal gyrus (STG), middle temporal gyrus (MTG), right DLPFC, left rostrolateral prefrontal cortex (RLPFC), left DMPFC, right pre‐supplementary motor area (pre‐SMA), and left anterior middle cingulate cortex (aMCC) were activated during the down‐negative condition (Table 3 and Figure 3). In addition, the right VLPFC, left insula, DLPFC, and DMPFC were activated during the up‐negative condition (Table 3 and Figure 4).

**TABLE 3:** Brain activation and deactivation for down‐ and up‐regulation.

Brain region	BA	Peak MNI coordinate			Z‐score	Cluster size
		X	Y	Z		
Down > look						
L Inf. Frontal G./VLPFC	47	−52	22	−12	5.64	1663
L Sup. Temporal G.	38	−46	14	−26	5.24	
L Precentral G./VLPFC	44	−52	12	2	5.13	
R Inf. Frontal G./VLPFC	47	42	26	−14	4.75	377
R Mid. Temporal G.	21	64	−18	−10	4.32	368
L Mid. Temporal G.	21	−48	−40	−2	5.02	845
R Inf. Frontal G./VLPFC	44	52	20	6	4.87	172
R Mid. Frontal G./DLPFC	8	50	16	40	4.50	883
L Sup. Frontal G./RLPFC	10	−18	54	16	5.98	388
R Sup. Temporal G.	39	50	−54	32	4.14	367
L Sup. Temporal G.	39	−42	−58	30	4.73	946
L Medial Frontal G./DMPFC	8	−6	32	46	6.13	3772
R Sup. Frontal G./pre SMA	6	14	24	56	5.56	
L Cingulate G./aMCC	32	−6	24	40	5.26	
Up>look						
R Inf. Frontal G./VLPFC	47	48	26	−6	4.12	181
L Inf. Orbital Frontal G./Insula	13	−28	32	12	3.82	335
L Inf. Frontal G./DLPFC	9	−54	22	20	4.11	375
L Sup. Frontal G./DMPFC	8	−4	30	52	4.16	546

*Note*: The threshold was set at *p < *0.05 FWE corrected (initial *p < *0.001 uncorrected).

Abbreviations: BA = Brodmann area; G. = gyrus; Inf. = inferior; L = left; Mid. = middle; MNI = Montreal Neurological Institute; R = right; Sup. = superior.

**FIGURE 3:**
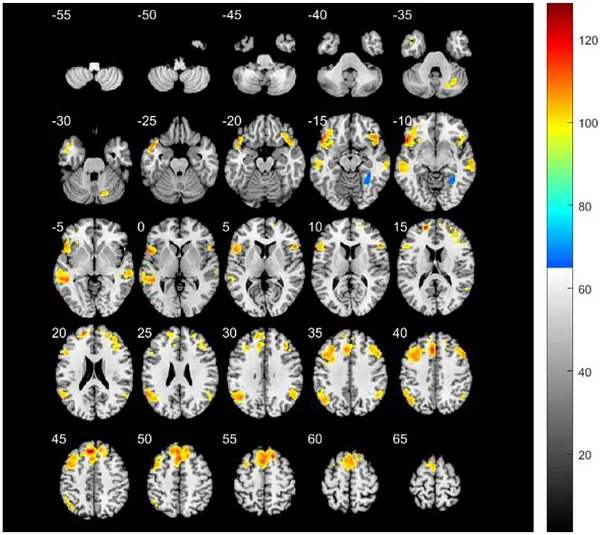
The activation results of down‐negative versus look‐negative condition. The threshold was set at *p < *0.05 FWE corrected (initial *p < *0.001 uncorrected).

**FIGURE 4:**
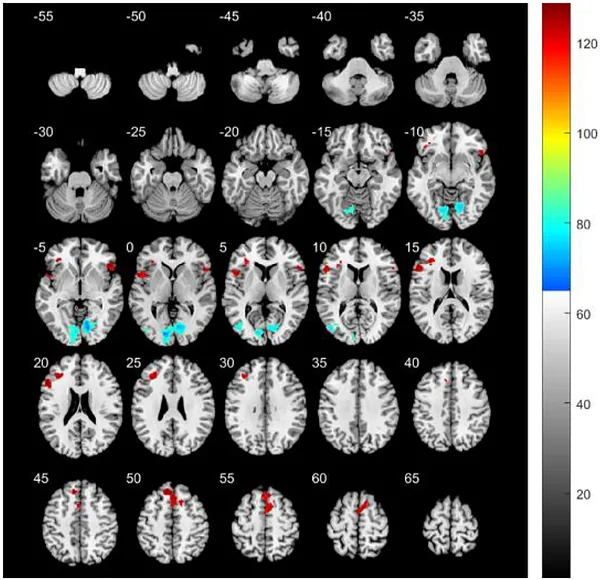
The activation results of up‐negative versus look‐negative condition. The threshold was set at *p < *0.05 FWE corrected (initial *p < *0.001 uncorrected).

Furthermore, the conjunction‐null analysis of up‐ versus look‐negative and down‐ versus look‐negative revealed that both up‐ and down‐regulation commonly activated left‐sided regions including the VLPFC, DLPFC, inferior parietal lobule, insula, and superior temporal gyrus, as well as the medial frontal cortex, comprising the DMPFC, ACC, and SMA (Table 4 and Figure 5). The up‐ versus down‐negative contrast revealed activation in the left postcentral gyrus, whereas the down‐ versus up‐negative contrast revealed activation in the bilateral lingual gyrus, left inferior occipital gyrus, right middle occipital gyrus, and right DLPFC (Table 4 and Figure 6).

**TABLE 4:** Common and differential activation of down‐ and up‐regulation.

Brain region	BA	Peak MNI coordinate			Z‐score	Cluster size
		X	Y	Z		
Conjunction						
L Inf. Frontal G./VLPFC/Insula/ Sup. Temporal G.	44/45/13/38	−50	12	4	4.83	757
L Inf. Parietal Lobule/ Supramarginal G./Angular G.	40/39	−60	−50	28	4.15	372
L Mid. Frontal G./DLPFC	9	−30	8	40	4.36	506
Medial Frontal G./DMPFC/ SMA/ACC	6/8/32	0	22	52	5.16	1210
Up > down						
L Postcentral G.	3	−46	−14	46	4.47	245
Down > up						
L Lingual G.	18	−4	−84	−8	4.69	495
R Lingual G.	18	10	−82	−2	4.31	221
L Inf Occipital G.	19	−46	−78	2	4.57	192
R Mid Occipital G.	37	44	−68	2	4.37	619
R Mid. Frontal G./DLPFC	8	44	30	40	4.54	456

*Note*: The threshold was set at *p < *0.05 FWE corrected (initial *p < *0.001 uncorrected).

Abbreviations: BA = Brodmann area; G. = gyrus; Inf. = inferior; L = left; Mid. = middle; MNI = Montreal Neurological Institute; R = right; Sup. = superior.

**FIGURE 5:**
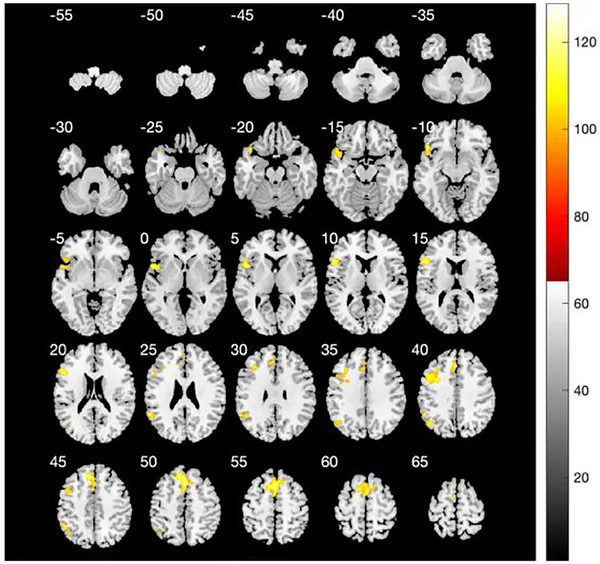
The conjunction‐null analysis results of up‐negative and down‐negative condition. The threshold was set at *p < *0.05 FWE corrected (initial *p < *0.001 uncorrected).

**FIGURE 6:**
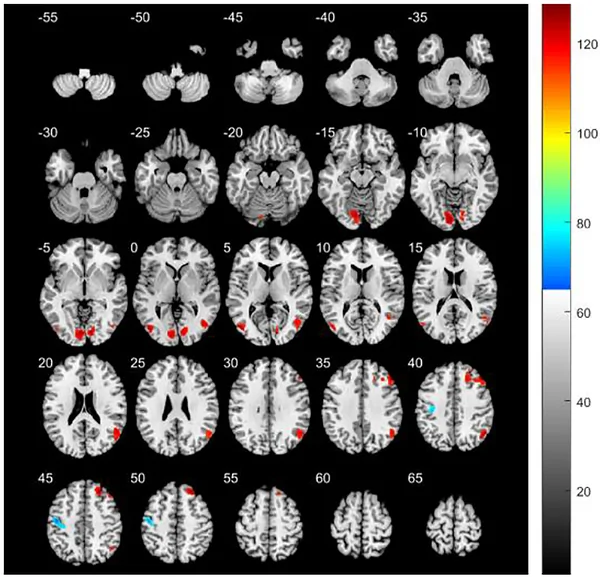
The activation results of down‐negative versus up‐negative condition. The threshold was set at *p < *0.05 FWE corrected (initial *p < *0.001 uncorrected).

### 3.3 Generalized Psychophysiological Interaction Analysis

The gPPI results are reported at an exploratory threshold of *p < *0.001 (uncorrected, k > 20) and are considered hypothesis‐generating. During down‐negative relative to the look‐negative condition, significant positive coupling was observed between the seed regions and the following target areas (Table 5 and Figure 7). The left VLPFC showed positive coupling with the right MTG and left middle occipital gyrus. The right VLPFC exhibited positive coupling with the right anterior cingulate gyrus and middle temporal gyrus. The right DLPFC showed positive coupling with the precentral gyrus. The left RLPFC showed positive coupling with the right superior and middle temporal gyri.

**TABLE 5:** The gPPI results for seed regions derived from the down‐negative versus look‐negative contrast.

Brain region	BA	Peak MNI coordinate			Z‐score	Cluster size
		X	Y	Z		
L Inf. Orbital Frontal G./VLPFC						
Down > look						
R Mid. Temporal G.	22/39	60	−58	8	3.85	39
L Mid. Occipital G.	19/37/39	−50	−70	6	3.84	28
R Inf. Orbital Frontal G./VLPFC						
Down > look						
R Anterior Cingulate G.	32	18	26	−8	4.31	77
R Mid. Temporal G.	21/22	58	−48	6	3.79	25
R Mid. Frontal G./DLPFC						
Down > look						
R Inf. Frontal G./precentral G./DLPFC	9	48	10	38	3.81	20
L Sup. Frontal G./RLPFC						
Down > look						
R Sup. Temporal G./Mid. Temporal G.	22/39	60	−58	18	4.04	25

*Note*: The threshold was set at *p < *0.001 uncorrected with cluster size k > 20 voxels.

Abbreviations: BA = Brodmann area; G. = gyrus; Inf. = inferior; L = left; Mid. = middle; MNI = Montreal Neurological Institute; R = right; Sup. = superior.

**FIGURE 7:**
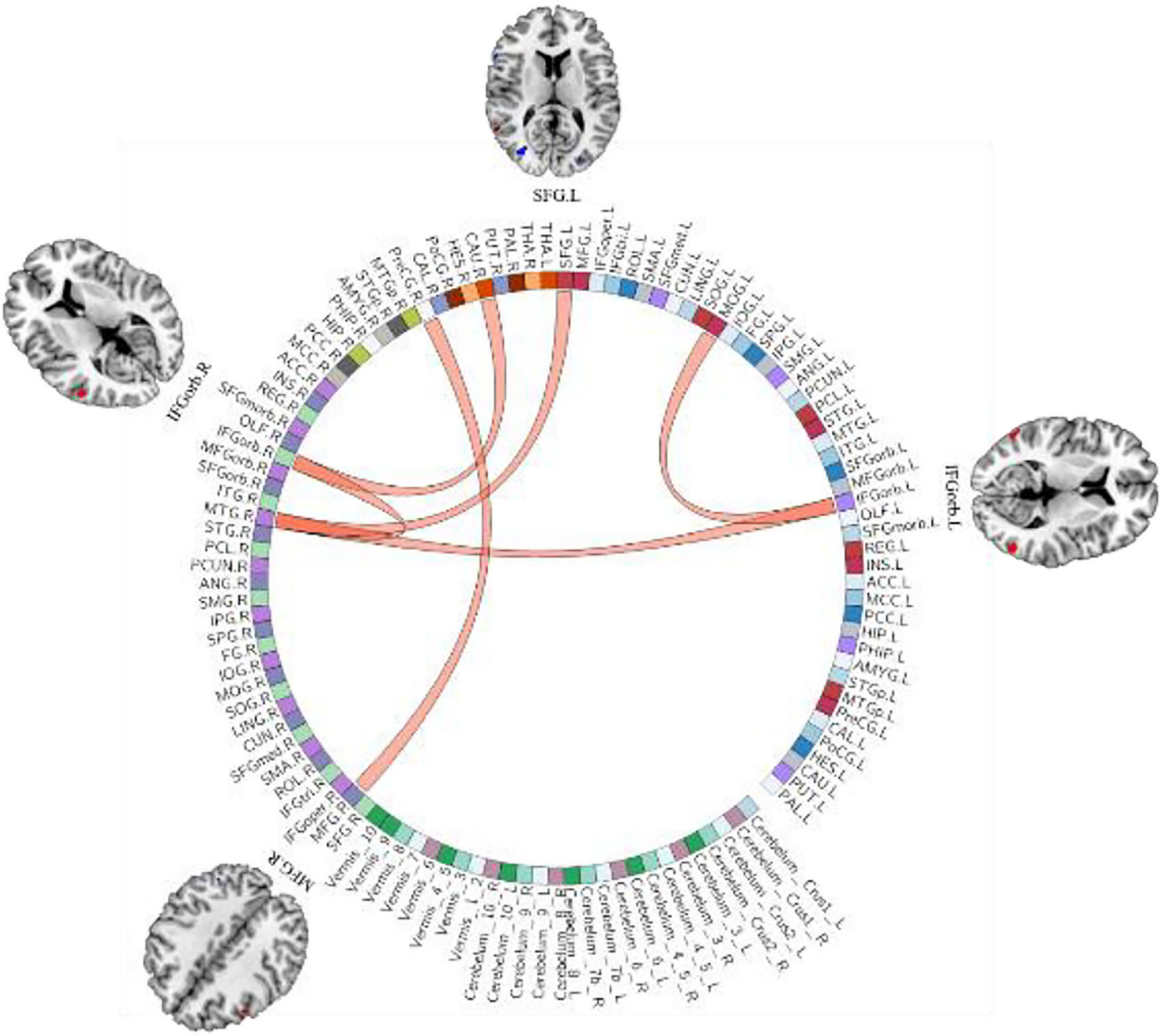
The gPPI connectivity map for seed regions in the down‐negative vs. look‐negative contrast. The results were assessed at a combined threshold of *p < *0.001, uncorrected, with a minimum cluster size of 20 voxels (L = left; R = right).

During up‐negative relative to the look‐negative condition, significant positive coupling was observed between the seed regions and the following target areas (Table 6 and Figure 8). The left DLPFC showed positive coupling with the left putamen, right parahippocampal gyrus, left orbitofrontal cortex, and insula. The left DMPFC showed positive coupling with the left insula. No significant clusters survived cluster‐level FWE correction in any of the tested contrasts.

**TABLE 6:** The gPPI results for seed regions derived from the up‐negative versus look‐negative contrast.

Brain region	BA	Peak MNI coordinate			Z‐score	Cluster size
		X	Y	Z		
L Triangular Inf. Frontal G./DLPFC						
Up > look						
L Putamen		−16	4	−8	3.98	23
L Lentiform Nucleus/Pallidum		−14	2	10	3.92	44
R Parahippocampa G.	28	16	−6	−14	3.89	24
L Inf. Orbital Frontal G. /IOFC	47	−38	30	−6	3.69	22
L Insula	13	−40	8	−10	3.57	26
L Putamen		−24	8	12	3.49	20
L Sup. Medial Frontal/DMPFC						
Up > look						
L Insula	13	−40	12	−6	3.57	36

*Note*: The threshold was set at *p < *0.001 uncorrected with cluster size k > 20 voxels.

Abbreviations: BA = Brodmann area; G. = gyrus; Inf. = inferior; L = left; Mid. = middle; MNI = Montreal Neurological Institute; R = right; Sup. = superior.

**FIGURE 8:**
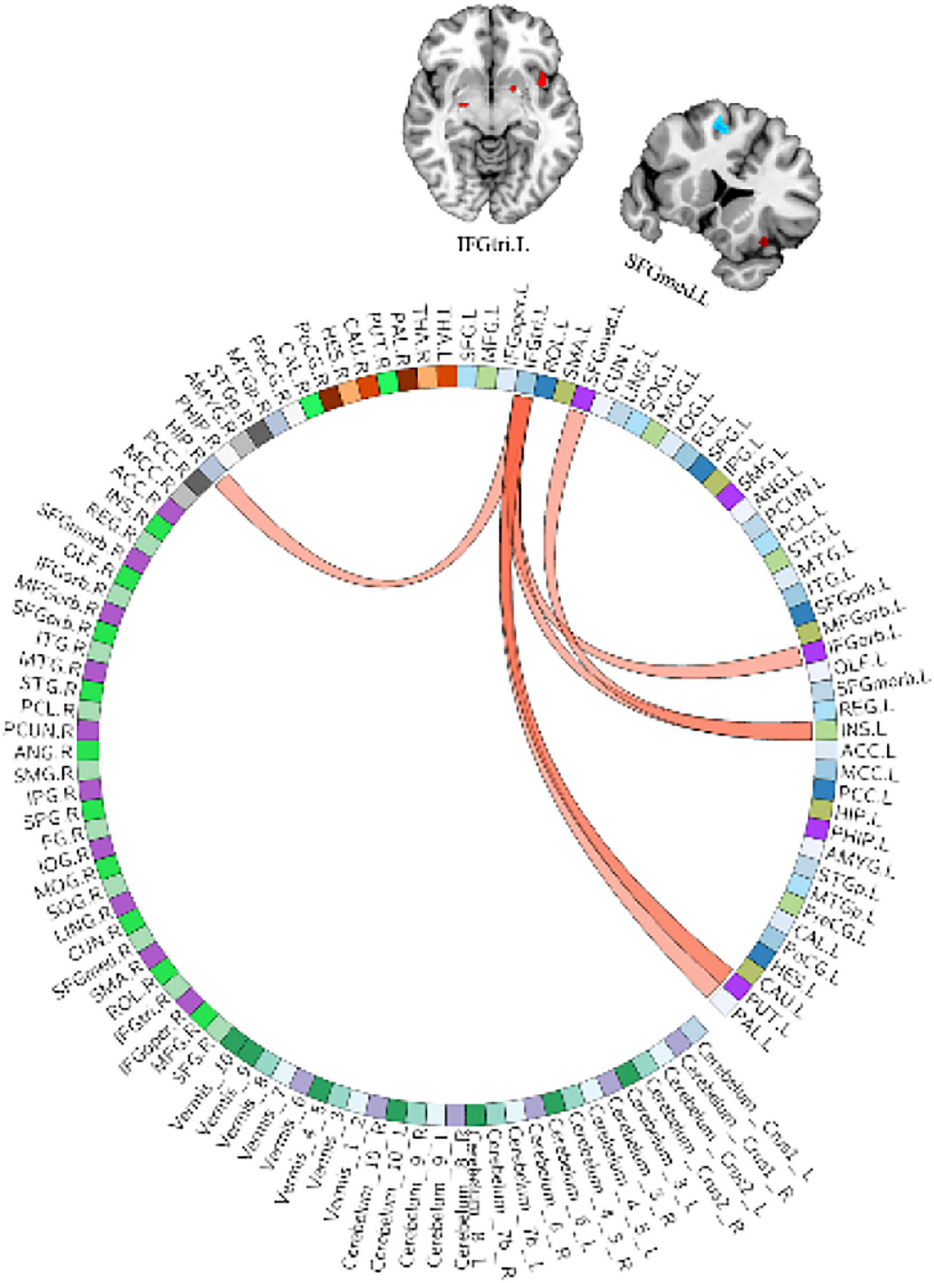
The PPI connectivity map for seed regions in the up‐negative versus look‐negative contrast. The results were assessed at a combined threshold of *p < *0.001, uncorrected with a minimum cluster size of 20 voxels (L = left; R = right).

## 4 Discussion

The present study combined whole‐brain activation and gPPI connectivity analyses to investigate the neural correlates of up‐ and down‐regulation of negative emotion. The conjunction‐null analysis revealed that both regulatory goals commonly engaged a predominantly left‐lateralized frontoparietal–medial frontal network, whereas the differential contrasts revealed goal‐specific activation patterns. The exploratory and uncorrected gPPI findings further suggested potential differences in coupling patterns with subcortical and temporal regions. By directly comparing the two regulation goals within the same cohort, this study provides a more comprehensive account of their shared and distinct neural correlates at both activation and connectivity levels.

Down‐regulation of negative emotion recruited bilateral prefrontal and temporal regions, including the VLPFC, DLPFC, and MTG, consistent with previous meta‐analyses of cognitive reappraisal (Morawetz et al. 2017; Buhle et al. 2014). Notably, the exploratory and uncorrected gPPI analysis further suggested enhanced coupling of the bilateral VLPFC with the right MTG and the ACC during down‐regulation. While previous meta‐analyses have reported prefrontal–prefrontal coupling during reappraisal, the present exploratory gPPI analysis suggested enhanced coupling involving temporal regions in addition to prefrontal regions (Berboth and Morawetz 2021). This discrepancy may reflect differences in task design, as the meta‐analyses included both reappraisal and distraction tasks (Morawetz et al. 2017). This pattern may be consistent with the role of these regions in semantic selection and processing, which may align with the cognitive demands of reinterpreting emotional stimuli during down‐regulation (Spitsyna et al. 2006; Garcin et al. 2018; Schurz et al. 2021). However, as the present study did not directly assess semantic content, this interpretation remains tentative. At the network level, the suggested coupling of the VLPFC with the ACC, core nodes of the control network, and with the MTG, which has been associated with semantic processing and interacts with default mode network regions, may indicate that down‐regulation involves coordination between prefrontal control systems and temporal regions supporting semantic and contextual retrieval. This interpretation aligns with recent findings that inter‐network connectivity distinguishes reappraisal from passive viewing (Horner et al. 2025).

Up‐regulation recruited the right VLPFC, left DLPFC, DMPFC, and insula. These findings are consistent with previous meta‐analyses showing that up‐regulation engages prefrontal regions (Morawetz et al. 2017). The exploratory and uncorrected gPPI analysis further suggested enhanced coupling between the left DLPFC and both the insula and the dorsal striatum, as well as between the left DMPFC and the insula, during up‐regulation. However, due to the limited number of PPI studies on up‐regulation, direct comparisons with our connectivity findings are currently not feasible. Functionally, the dorsal striatum, as part of the cortico–striatal circuitry, has been implicated in integrating cognitive signals with reward‐related outcomes, while the insula is involved in interoception and emotional awareness (Zhang et al. 2024; Haber and Knutson 2010; Feinstein et al. 2020). Given the role of the dorsal striatum in integrating cognitive signals with motivational and affective outcomes, these suggested coupling patterns may reflect the recruitment of incentive‐related or outcome‐anticipation circuits during up‐regulation. The suggested DLPFC–insula and DMPFC–insula coupling may reflect increased emotional awareness, consistent with the demands of amplifying subjective emotional experience. At the network level, these exploratory findings may suggest that upregulation relies primarily on interactions of the cognitive control network with reward‐related networks and salience networks. Together with the findings for down‐regulation, these results indicate that down‐regulation may rely more on cognitive re‐evaluation through semantic processing, whereas up‐regulation may rely more on reward‐related and interoceptive mechanisms.

The conjunction‐null analysis revealed common activation in a predominantly left‐lateralized frontoparietal–medial frontal network, mainly involving the left VLPFC, DLPFC, and inferior parietal lobule, as well as the medial frontal cortex (DMPFC, ACC, and SMA). The activation of the DLPFC, VLPFC, DMPFC, and SMA is consistent with previous findings (Morawetz et al. 2017; Holland and Kensinger 2013). These regions have been consistently implicated in cognitive control and semantic processing during cognitive reappraisal, with the DMPFC and SMA additionally supporting emotional appraisal and response preparation (Morawetz et al. 2017; Buhle et al. 2014; Kohn et al. 2014; McRae et al. 2010). In addition, the present conjunction‐null analysis identified the left inferior parietal lobule and ACC, which were not reported in the previous meta‐analytic conjunction. These additional regions may partly reflect methodological differences between the conjunction‐null approach and the previous ALE meta‐analysis. Functionally, the left inferior parietal lobule may support attentional reorientation and semantic integration during reappraisal, whereas the ACC may contribute to conflict monitoring and response selection (Morawetz et al. 2016; Fang et al. 2024; Lee et al. 2021). Together, these findings suggest that up‐ and down‐regulation share a core emotion‐regulation network involving prefrontal cognitive control, parietal attentional and semantic processing, and medial frontal conflict monitoring and response preparation.

However, despite these shared activations, up‐ and down‐regulation also showed distinct activation patterns. Consistent with previous meta‐analyses, the right DLPFC was specifically engaged during down‐regulation, supporting its role in cognitive control and goal maintenance during reappraisal (Morawetz et al. 2017). While the postcentral gyrus is primarily associated with somatosensory processing, its specific role in emotion regulation remains unclear (Kropf et al. 2019). Its activation during up‐regulation may relate to increased bodily attention or somatomotor engagement, though this interpretation awaits confirmation with psychophysiological measures (Kropf et al. 2019; Min et al. 2022; Dretsch et al. 2019). In addition, down‐regulation recruited occipital regions, including the bilateral lingual gyrus, which may reflect increased visual and perceptual processing (Palejwala et al. 2021). These distinct patterns suggest that the two regulatory goals engage partially different neural substrates beyond their shared frontoparietal–medial frontal network.

Unlike previous studies that reported prefrontal‐amygdala coupling during reappraisal, our study did not find significant amygdala activation. Previous mediation studies have demonstrated that effective reappraisal involves a PFC–VMPFC–amygdala pathway, in which prefrontal regions modulate amygdala responses indirectly through the VMPFC (Urry et al. 2006; Johnstone et al. 2007; Ochsner et al. 2012). In the present study, VMPFC was not significantly activated, consistent with previous findings (Buhle et al. 2014). This may account for the absence of amygdala modulation, as VMPFC may serve as a critical relay between prefrontal control regions and the amygdala. Importantly, the absence of amygdala activation does not necessarily imply that reappraisal was ineffective. It remains possible that reappraisal‐related amygdala modulation was present but not detected due to limited statistical power or the absence of a priori region‐of‐interest (ROI) analysis. Alternatively, some reappraisal effects may occur through amygdala‐independent routes, as suggested by previous work (Buhle et al. 2014; Yoon and Jung 2025; Doré et al. 2017). However, such pathways could not be confirmed in the present data. Therefore, while the present null result does not rule out amygdala involvement, the specific pathways recruited during reappraisal may vary with task design and individual differences. These findings should not be taken as evidence against the amygdala's role in emotion regulation.

The present study extends previous findings in two ways. First, by directly comparing up‐ and down‐regulation within the same cohort, we identified both shared and goal‐specific activation patterns, with the shared activation converging on a frontoparietal–medial frontal network. Second, the exploratory and uncorrected gPPI analysis suggested that the two regulatory goals may differ in network‐level coupling, although these connectivity findings require replication. These results contribute to a growing body of literature advocating a network‐based perspective on emotion regulation, and highlight the importance of considering both activation and connectivity when characterizing emotion regulation processes.

### 4.1 Limitations

Although the present study provides comprehensive activation and gPPI analyses, it has several limitations. First, the modest sample size (*n = *21) was determined by feasibility, and no a priori power analysis was conducted. This may have limited statistical precision and increased the risk of Type II errors and inflated effect‐size estimates, particularly for subcortical regions with lower signal‐to‐noise ratios. Although the sample size is comparable to several previous task‐fMRI studies of cognitive reappraisal, the reliability of the present results should be confirmed in future studies with larger cohorts. Second, correction strategies differed between the activation and connectivity analyses. Whole‐brain activation results were corrected using cluster‐level FWE (*p < *0.05), whereas gPPI results were explored at a more liberal threshold (*p < *0.001, uncorrected, *k* > 20) without correction across seeds or contrasts. This liberal threshold may increase the risk of false‐positive findings. Therefore, all gPPI results should be interpreted as exploratory and hypothesis‐generating, and require independent replication. Third, we did not collect self‐reported measures of task compliance after scanning. Although participants were instructed to follow the regulation cues, and those who failed to respond on more than five trials were excluded, the absence of a formal post‐scan compliance rating limits our ability to fully assess participants’ engagement with the task. Future studies may benefit from including such measures to better account for individual differences in task performance.

## 5 Conclusions

In summary, the present study revealed three main findings. First, both up‐ and down‐regulation commonly engaged a predominantly left‐lateralized frontoparietal–medial frontal network, indicating a shared regulatory core across regulatory goals. Second, at the activation level, down‐regulation specifically recruited the right DLPFC and occipital regions, whereas up‐regulation specifically recruited the left postcentral gyrus, suggesting that the two regulatory goals engage partially distinct neural substrates. Third, the exploratory and uncorrected gPPI analysis suggested that down‐regulation was associated with prefrontal–temporal coupling, whereas up‐regulation was associated with prefrontal–striatal and prefrontal–insula coupling. These findings suggest that cognitive reappraisal may rely on a common frontoparietal–medial frontal network, but up‐ and down‐regulation may be supported by different patterns of task‐modulated functional connectivity. Given the exploratory nature of the gPPI findings and the modest sample size, these results should be considered preliminary and require replication in larger independent samples.

## Author Contributions

**Yuluo Liu**: conceptualization, funding acquisition, data curation, formal analysis, Writing – original draft, Writing – review and editing. **Shan Gui**: data curation. **Longda Ma**: data curation, formal analysis. **Lian Yang**: resources. **Zilong Liu**: resources. **Yiwu Zhou**: funding acquisition, validation, writing – original draft. **Xiangyu Wang**: conceptualization, validation, writing – original draft, writing – review and editing. All the authors approved the final version to be published and agreed to be accountable for the integrity and accuracy of all aspects of the work.

## Funding

Funding for this study was provided by the Youth Innovative Talents Program in Guangdong Province's Ordinary Universities (2024KQNCX124) and the Strong Teacher Project of Guangdong Police College (2025FY12).

## Ethics Statement

The study was approved by the Human Ethics Committee for Human Research in Tongji Medical College.

## Consent

All participants provided written informed consent before the experiment and were paid for their participation. The author confirms that the work described has not been published previously, and not under consideration for publication elsewhere, in whole or in part. And its publication has been approved by all co‐authors, if any. Moreover, its publication has been approved (tacitly or explicitly) by the responsible authorities at the institution where the work is carried out.

## Conflicts of Interest

The authors declare no conflicts of interest.

## Data Availability

The datasets generated and analyzed during the current study are not publicly available because they constitute an excerpt of research in progress, but are available from the corresponding author upon reasonable request.
